# Computer Simulation of Anisotropic Polymeric Materials Using Polymerization-Induced Phase Separation under Combined Temperature and Concentration Gradients

**DOI:** 10.3390/polym11061076

**Published:** 2019-06-21

**Authors:** Shima Ghaffari, Philip K. Chan, Mehrab Mehrvar

**Affiliations:** Department of Chemical Engineering, Ryerson University, 350 Victoria Street, Toronto, ON M5B 2K3, Canada; shima.ghaffari@ryerson.ca (S.G.); mmehrvar@ryerson.ca (M.M.)

**Keywords:** polymerization-induced phase separation, anisotropic morphology, temperature gradient, concentration gradient, spinodal decomposition

## Abstract

In this study, the self-condensation polymerization of a tri-functional monomer in a monomer-solvent mixture and the phase separation of the system were simultaneously modeled and simulated. Nonlinear Cahn–Hilliard and Flory–Huggins free energy theories incorporated with the kinetics of the polymerization reaction were utilized to develop the model. Linear temperature and concentration gradients singly and in combination were applied to the system. Eight cases which faced different ranges of initial concentration and/or temperature gradients in different directions, were studied. Various anisotropic structural morphologies were achieved. The numerical results were in good agreement with published data. The size analysis and structural characterization of the phase-separated system were also carried out using digital imaging software. The results showed that the phase separation occurred earlier in the section with a higher initial concentration and/or temperature, and, at a given time, the average equivalent diameter of the droplets <*d*_ave_> was larger in this region. While smaller droplets formed later in the lower concentration/temperature regions, at the higher concentration/temperature side, the droplets went through phase separation longer, allowing them to reach the late stage of the phase separation where particles coarsened. In the intermediate stage of phase separation, <*d*_ave_> was found proportional to $t^{*\alpha }$, where α was in the range between $\frac{1}{3}$ and $\frac{1}{2}$ for the cases studied and was consistent with published results.

## 1. Introduction

Polymerization-induced phase separation (PIPS) is a practical method of fabricating functional polymeric materials with symmetric structures and has been widely studied numerically and experimentally [1,2,3,4,5]. Isotropic polymeric materials formed by the off-critical conventional PIPS method consist of homogeneous-sized droplets which are uniformly distributed in the polymer matrix. Such polymeric products have numerous engineering applications such as biomedical materials [6], porous thermosets [7], nanocomposite materials [8,9,10], and polymer-dispersed liquid crystals [11].

The functionalities and structural properties of polymeric materials can be modified by imposing external forces to a polymer solution undergoing phase separation to form non-uniform anisotropic microstructures. Anisotropic porous polymer membranes [12,13,14,15] and switchable holographic polymer-dispersed liquid crystal films [16] are some practical examples of anisotropic phase separated polymeric materials. Asymmetry can be generated by applying, singly or in combination, an external force like shear flow [17], an electric field [18,19], a surface effect [20,21,22,23,24], a controlled chemical reaction [25], a concentration gradient [14,15,26,27], or a temperature gradient [12,13,15,28].

Several modeling and experimental studies have been carried out on the fabrication of anisotropic polymeric materials by the thermal-induced phase separation (TIPS) technique. Caneba and Soong [12,13] investigated the formation of an anisotropic membrane with graded pore sizes using the thermal-inversion method. They experimentally and numerically studied the binary system of poly(methyl methacrylate) (PMMA)/sulfolane as a polymer/solvent undergoing the thermal casting procedure; heat was removed from one face of the sample while the other face was insulated. Phase separation was first induced in the cooler layer of the solution before being propagated into the hotter layers. For the initial compositions of 10 wt% and 15 wt% PMMA, the asymmetric morphology consisted of small pore sizes near the conducting medium and large pore sizes near the insulating surface was observed. The average pore size for the initial composition of 10 wt % PMMA was obtained larger than that for 15 wt% PMMA. Matsuyama et al. [14,15] produced a membrane with asymmetric morphology by the thermal-induced phase separation of isotactic polypropylene (iPP)/diphenyl ether solution; a polymer concentration gradient, singly and along with a temperature gradient, was induced in the solution before the phase separation occurred. The diluent was partially evaporated from the top side of the sample to establish the concentration gradient. Larger pores were observed at the bottom of the membrane where the polymer concentration was lower, while smaller pores were formed at the top layer in which the polymer was more concentrated. Combining the temperature gradient obtained by putting the top surface of the sample in the cold water after evaporation with concentration gradient, they found a noticeable asymmetric structure with a thin dense skin layer at the top of the membrane and a thick porous layer at the bottom. Smaller pores at the lower temperature region were observed. Tran-Cong and Okinaka [28] examined the TIPS of poly(2-chlorostyrene)/poly(vinyl methyl ether) (P2CS/PVME) blends under a temperature gradient. The average initial concentration of the sample was set as a critical concentration, and the temperature gradient was defined in a range from above the critical temperature to below the critical temperature. Droplet-type morphology was obtained at the high temperature side of the sample as phase separation reached the late stage, while the interconnected morphology was found in the low temperature region. The average diameter of the droplets versus time followed power law $d\propto t^{\alpha }$, where α varied from 0.3 to 0.44 through the temperature gradient.

Lee et al. [29,30] developed one-dimensional and two-dimensional models to investigate the thermal-induced phase separation of polymer solutions via spinodal decomposition using the nonlinear Cahn–Hilliard and Flory–Huggins theories. A spatial temperature gradient was considered in the models to induce an anisotropic structure. The results showed that droplets first appeared at the low-temperature region of the system and later at the high-temperature domain. A higher density of the droplets and a greater average equivalent diameter were found in the lower-temperature sections of the mixture. The circularity of the solvent-reach droplets was independent to the temperature/location. Chan [31] simulated the TIPS mechanism of a polymer/solvent mixture by imposing a linear concentration gradient. The results were consistent with published experimental works. Increasing the initial concentration of the solvent along the membrane led to the formation of larger particles as well as the decrease of phase separation process time. Jiang and Chan [32] provided a two-dimensional model to determine the effect of the initial concentration of a solvent on the structural feature of polymer/solvent mixtures located at three different regions of the binary phase diagram while undergoing the TIPS process. For the sample located at the left (right) side the critical region, phase separation occurred earlier at the higher (lower) initial concentration edge; in addition, larger solvent-rich (polymer-rich) droplets were observed in this part of the phase-separated mixture. In the third sample, in which the concentration gradient passed the critical concentration, a combination of interconnected and droplet-type morphologies was observed. Hong and Chan [33] mathematically investigated the effect of simultaneous temperature and concentration gradients on the TIPS process where the linear gradients were applied along the vertical direction in the same and opposite directions. For the case of same direction gradients, particles first grew at the top and bottom edges (the high concentration and low temperature layers, respectively), which then propagated inward. The average size was found larger at both the top and bottom sides, while a smaller average size was found in the middle layers. Applying the gradients in opposite directions, however, led to the production of larger particles first at the top edge (at the high concentration and low temperature side) and smaller ones in the lower layers. Tabatabaieyazdi et al. [22,23,24] modeled short-range, long-range, and multiple surface-directed thermal-induced phase separation phenomena of polymer blends. According to their method, phase separation proceeded while, simultaneously, surfaces preferentially attracted one of the components of the blends. A linear temperature gradient was also applied perpendicular to the surfaces. For the short-range surface effect, they found the growth rate of the enrichment layer was faster at the early stage of phase separation, but it was slower at the intermediate stage. Structural anisotropy was obtained and included wetting layers on or near the surfaces and droplet-type/interconnected morphology in the bulk.

In contrast, there have only been a few studies carried out on the fabrication of anisotropic polymeric materials by the PIPS method. Oh and Rey [34] utilized a 2-D computational model to investigate the polymerization-induced phase separation of a monomer/polymer mixture which had an upper critical solution temperature (UCST) in the presence of a linear temperature gradient in the vertical direction. Droplets first appeared in the high temperature region as it was thrust into the phase-separating region of the binary phase diagram first. At the low temperature side, a lamella (interconnected cylinder-type) structure was observed where a strong (weak) temperature gradient was imposed.

A spatial anisotropic structure of inhomogeneous polymers introduced by means of the PIPS phenomenon under a linear temperature gradient was modeled in a 1-D domain by Lee et al. [35]. The model demonstrated that the total process induction time decreased as the temperature increased along the sample. Consequently, particles grew earlier at the high temperature side and then propagated toward lower temperature sections. The characteristic length was a complicated function of the stage of phase separation, the rate of phase separation, and the polymerization reaction.

Fujiki et al. [36] reviewed the fabrication of spatially graded polymer blends with enhanced mechanical and physical properties through photopolymerization using a UV light intensity gradient. Based on their method, functionalized gradient polymer blends were fabricated by irradiation from one side of the sample while photopolymerization proceeded.

Based on the authors’ knowledge of published studies, there is no computer simulation study on the morphology development and characteristics of anisotropic polymeric mixtures fabricated by the PIPS mechanism under an initial concentration gradient singly and in combination with a temperature gradient using the C–H theory. This computer simulation study forms the basis of this manuscript.

## 2. Model Development

Phase separation by spinodal decomposition is initiated by thermal concentration fluctuations in the unstable region. The phase separation undergoes through three stages. In the early stage, the concentration fluctuations increase in magnitude while the characteristic length scale remains constant. Both the concentration fluctuations and characteristic length scale increase in magnitude during the intermediate stage. Lastly, in the late stage, only the characteristic length scale increases in magnitude since the concentration fluctuations have reached the equilibrium value.

In the current section, a two-dimensional model incorporating linear concentration and/or temperature gradients is developed in a square domain for the phase separation of a monomer/solvent system induced by the self-condensation polymerization of the monomer. The nonlinear Cahn–Hilliard theory, which describes phase separation by spinodal decomposition based on the continuity equation, is utilized [37]. Based on the C–H theory, the total free energy of the heterogeneous binary solution is considered as:

(1) $$F=\int \left[f\left(c\right)+\kappa {\left(\nabla c\right)}^{2}\right]dV$$

where f(c) is the free energy density of the homogeneous solution, κ is the interfacial energy coefficient, and c is the concentration of the solvent, which is defined as the volume fraction in this paper. The Flory–Huggins free-energy equation defines f(c) as [38]:

(2) $$f(c)=\frac{k_{B}T}{\nu }\left[\frac{c}{N_{1}}\ln c+\frac{\left(1-c\right)}{N_{2}}\ln(1-c)+\chi c(1-c)\right]$$

where $k_{B}$ is Boltzmann’s constant, T is the temperature, ν is the volume of a cell, χ is Flory’s interaction parameter, and $N_{1}$ and $N_{2}$ are the degrees of polymerization of the solvent and monomer, respectively. In this study, it was considered that $N_{1}=1$. Though the Flory’s interaction parameter is related to the concentration, temperature, and degree of polymerization, it can be expressed as a function of temperature for simplicity [39]:

(3) $$\chi =\frac{1}{2}-\psi [1-\frac{\theta }{T}]$$

where θ is the theta temperature and ψ is a dimensionless entropy of dilution parameter. Imposing Equation (1) into the continuity equation, the C–H equation is derived as follows to predict the dynamic behavior of the phase separation process [40,41]:

(4) $$\frac{\partial c}{\partial t}=\nabla \cdot [M\nabla [\frac{\partial f}{\partial c}-2\kappa \nabla^{2}c]]$$

where the mobility, M, is a function of molecular weights and concentrations of the components. Using slow-mode theory [42], the mobility is expressed as:

(5) $$\frac{1}{M}=\frac{1}{M_{1}}+\frac{1}{M_{2}}$$

The self-mobilities of the solvent and solute, $M_{1}$ and $M_{2}$, respectively, in turn depend on the self-diffusivities:

(6) $$D_{i}=M_{i}(\frac{\partial^{2}f}{\partial c_{i}{}^{2}})$$

The self-diffusion coefficient of each component ($D_{i}$) is expressed by the Rouse law [42]. The Rouse theory ignores the impact of the entanglement of polymer chains, which is reasonable for short chain polymers where $N_{i}$ < 200 and relates the self-diffusion coefficient of each component to its degree of polymerization:

(7) $$D_{i}=\frac{k_{B}T}{\xi_{i}N_{i}}\;\mathrm{for}\;i=1,\;2$$

where $\xi_{i}$ is the frictional coefficient per cell of the solvent or polymer molecule. Assuming the frictional coefficients of the polymer and solvent cells are the same ($\xi_{1}$ = $\xi_{2}$ = ξ) and do not depend on the concentrations and temperature, the mobility is finally obtained as [42]:

(8) $$M=\frac{\nu c(1-c)}{\xi }$$

The interfacial energy coefficient is proportional to the molecular weight of the polymer and can be related to the degree of polymerization [1]:

(9) $$\kappa =\kappa_{0}N_{2}$$

where $\kappa_{0}$ is the interfacial energy coefficient of a linear polymer.

The constituent components of the mixture are a solvent with degree of polymerization of one and a tri-functional monomer undergoing self-condensation polymerization reaction. The self-condensation polymerization is a second order reaction, with its kinetic rate of reaction defined as [1]:

(10) $$\frac{dp}{dt}=k_{1}{\left(1-p\right)}^{2}$$

where p is the extent of reaction and $k_{1}$ is the rate constant which follows the Arrhenius equation:

(11) $$k_{1}=A\exp(\frac{-E_{a}}{RT})$$

where A, $E_{a}$, and R are the pre-exponential parameter, activation energy, and ideal gas rate constant, respectively. The extent of reaction can be analytically obtained from Equation (10):

(12) $$p=\frac{k_{1}t}{1+k_{1}t}.$$

$N_{2}$ is considered as the weight average degree of polymerization [1]:

(13) $$N_{2}=\frac{1+\alpha }{1-\alpha (f-1)}$$

where α is the branching coefficient which is equal to p (α =p) for this reaction of a single reactant and f is the functionality of the reactant which is equal to three ($A_{3}$). The degree of polymerization ($N_{2}$) is obtained as a function of time and temperature by combining Equations (11)–(13):

(14) $$N_{2}=\frac{1+2At\exp(\frac{-E_{a}}{RT})}{1-At\exp(\frac{-E_{a}}{RT})}$$

The linear initial concentration is imposed to the system in the x-direction as follows:

(15) $$c_{0}=(\frac{c_{02}-c_{01}}{x_{2}-x_{1}})(x-x_{1})+c_{01}$$

where $c_{01}$ and $c_{02}$ are the initial concentrations at positions $x_{1}$ and $x_{2}$, respectively. A linear temperature gradient can also be applied as follows:

(16) $$T=({\frac{T_{2}-T_{1}}{x_{2}-x}}_{1})(x-x_{1})+T_{1}$$

where $T_{1}$ and $T_{2}$ are the temperature at positions $x_{1}$ and $x_{2}$, respectively. All the parameters and variables are nondimensionalized in this paper and based on the following relations:

(17a) $$\mathrm{Dimensionless}\;\mathrm{length}\;l^{*}=\frac{l}{L}$$

(17b) $$\mathrm{Dimensionless}\;\mathrm{temperature}\;T^{*}=\frac{T}{\theta }$$

(17c) $$\mathrm{Dimensionless}\;\mathrm{concentration}\;c^{*}=c$$

(17d) $$\mathrm{Dimensionless}\;\mathrm{activation}\;\mathrm{energy}\;E_{a}^{*}=\frac{E_{a}}{R\theta }$$

(17e) $$\mathrm{Dimensionless}\;\mathrm{pre}-\mathrm{exponential}\;\mathrm{factor}\;A^{*}=\frac{AL\xi }{2\kappa_{0}\nu }$$

(17f) $$\mathrm{Dimensionless}\;\mathrm{time}\;t^{*}=\frac{2\nu \kappa_{0}t}{\xi L^{4}}$$

(17g) $$\mathrm{Dimensionless}\;\mathrm{diffusivity}\;D=\frac{k_{B}\theta L^{2}}{2\kappa_{0}\nu }$$

(17h) $$\mathrm{Dimensionless}\;\mathrm{rate}\;\mathrm{constant}\;K^{*}=A^{*}t^{*}\exp(\frac{-E_{a}^{*}}{T^{*}})$$

(17i) $$\mathrm{Dimensionless}\;\mathrm{degree}\;\mathrm{of}\;\mathrm{polymerization}\;N_{2}=\frac{1+2K^{*}}{1-K^{*}}$$

where L is the sample length. The dimensionless C–H equation is derived by inserting the above dimensionless parameters into the governing equations. For cases without a temperature gradient, the spatio–temporal concentration equation obtained by combining Equations (2), (4), (8), (9), and (17a–i) is expressed as:

(18) $$\begin{array}{l} \frac{\partial c^{*}}{\partial t^{*}}=DT^{*}[\frac{1}{N_{2}}-1-2\chi (1-2c^{*})]\nabla^{*}c^{*}.\nabla^{*}c^{*}+DT^{*}[1-c^{*}+\frac{c^{*}}{N_{2}}-2\chi c^{*}(1-c^{*})]\nabla^{*}{}^{2}c^{*}\\ -N_{2}(1-2c^{*})\nabla^{*}c^{*}\cdot \nabla^{*}{}^{3}c^{*}-N_{2}c^{*}(1-c^{*})\nabla^{*}{}^{4}c^{*} \end{array}$$

While for those cases facing a temperature gradient, the following partial differential equation is obtained by combining Equations (2), (4), (8), (9), (14), (16), and (17a–i):

(19) $$\begin{array}{l} \frac{\partial c^{*}}{\partial t^{*}}=D\left[\frac{c^{*}(1-c^{*})[\ln(1-c^{*})+1]\left(E_{a}^{*}fK^{*}\right)}{T^{*}{}^{2}(1+2K^{*})}\right]\nabla^{*}T^{*}\cdot \nabla^{*}T^{*}\\ +D\left[\frac{7}{2}-8c^{*}+4c^{*}{}^{2}-\psi +8\psi c^{*}(1-c^{*})+(1-2c^{*})\ln c^{*}-\frac{(1-2c^{*})\ln(1-c^{*})+1-4c^{*}}{N_{2}}\right]\nabla^{*}c^{*}\cdot \nabla^{*}T^{*}\\ +D\left[\frac{\left[(1-2c^{*})\ln(1-c^{*})+1-4c^{*}\right]\left(E_{a}^{*}fK^{*}\right)}{T^{*}(1+2K^{*})}\right]\left[\frac{E_{a}^{*}(1-2K^{*}+fK^{*})}{T^{*}(1+2K^{*}-fK^{*})}-\frac{2E_{a}^{*}fK^{*}}{T^{*}(1+2K^{*})(1+2K^{*}-fK^{*})}\right]\nabla^{*}c^{*}\cdot \nabla^{*}T^{*}\\ +DT^{*}\left[\frac{1}{N_{2}}-1-2\chi (1-2c^{*})\right]\nabla^{*}c^{*}\cdot \nabla^{*}c^{*}\\ +DT^{*}[1-c^{*}+\frac{c^{*}}{N_{2}}-2\chi c^{*}(1-c^{*})]\nabla^{*}{}^{2}c^{*}\\ -(1-2c^{*})\left(\frac{\partial N_{2}}{\partial T^{*}}\right)\nabla^{*}{}^{2}c^{*}\nabla^{*}c^{*}\cdot \nabla^{*}T^{*}\\ -c^{*}(1-c^{*})\left(\frac{\partial^{2}N_{2}}{\partial T^{*}{}^{2}}\right)\nabla^{*}{}^{2}c^{*}\nabla^{*}T^{*}\cdot \nabla^{*}T^{*}\\ -2c^{*}(1-c^{*})\left(\frac{\partial N_{2}}{\partial T^{*}}\right)\nabla^{*}T^{*}\cdot \nabla^{*}{}^{3}c^{*}\\ -N_{2}(1-2c^{*})\nabla^{*}c^{*}\cdot \nabla^{*}{}^{3}c^{*}\\ -N_{2}c^{*}(1-c^{*})\nabla^{*}{}^{4}c^{*} \end{array}$$

The initial condition is defined as the infinitesimal random concentration fluctuations existing in the homogeneous mixtures.

(20) $$c^{*}\left(t^{*}=0\right)=c_{0}{}^{*}+\delta c^{*}\left(t^{*}=0\right)$$

where $c_{0}{}^{*}$ is the dimensionless initial average concentration and $\delta c^{*}$ represents the infinitesimal dimensionless concentration fluctuations. The fluctuations are applied in the range of $\pm 10^{-6}$. The boundary conditions include, respectively, the natural and zero mass boundary conditions [40,41]:

(21) $$[\nabla^{*}c^{*}]\cdot n=0$$

**j** = 0 (22)

where **n** is the unit normal to the surface. For a two dimensional square domain, the natural boundary conditions become:

(23a) $$\frac{\partial c^{*}}{\partial x^{*}}=0,\;\mathrm{at}\;t^{*}>0,\mathrm{and}\;x^{*}=0\;\mathrm{and}\;x^{*}=1$$

(23b) $$\frac{\partial c^{*}}{\partial y^{*}}=0,\;\mathrm{at}\;t^{*}>0,\mathrm{and}\;y^{*}=0\;\mathrm{and}\;y^{*}=1$$

In addition, the zero mass flux boundary conditions are expressed as follow:

(23a) $$\frac{\partial^{3}c^{*}}{\partial x^{*}{}^{3}}+\frac{\partial^{3}c^{*}}{\partial x^{*}\partial y^{*}{}^{2}}=0,\;\mathrm{at}\;t^{*}>0,\mathrm{and}\;x^{*}=0\;\mathrm{and}\;x^{*}=1$$

(23b) $$\frac{\partial^{3}c^{*}}{\partial y^{*}{}^{3}}+\frac{\partial^{3}c^{*}}{\partial y^{*}\partial x^{*}{}^{2}}=0,\;\mathrm{at}\;t^{*}>0,\mathrm{and}\;y^{*}=0\;\mathrm{and}\;y^{*}=1$$

The Galerkin finite element method is used to solve the governing equations numerically. This method transforms the governing equations to a system of time-dependent ordinary differential equations which are solved using the Newton–Raphson method. The implicit Euler method is used for time integration. Convergence is assumed when the length of the vector of two successive computed solutions is less than 10^−6^. A mesh of 100 × 100 is used in the computer simulation. The computing code was written in C^++^ and executed on the Compute Canada, Sharcnet Consortium, Graham Resource (128G/32Core); each run took 10−18 h.

## 3. Results and Discussion

The results of the computer simulations are presented in this section. Though a comprehensive parametric study was performed, only eight cases are presented here, as they best reflect the objectives of this paper. The parametric study incorporated experimental values typically found in the literature for the length *L* [22], dimensionless diffusivity *D* [4], temperature *T* [14], time *t* [28], reaction rate constant *k*_1_ [43], and degree of polymerization [4]. The first part includes the results showing phase-separated structure and morphology development for the eight cases listed in Table 1. The second part shows the size analysis obtained for the Cases 1, 4, and 5 using ImageJ software [44].

### 3.1. Phase-Separated Structure and Morphology Development

**Figure 1:**
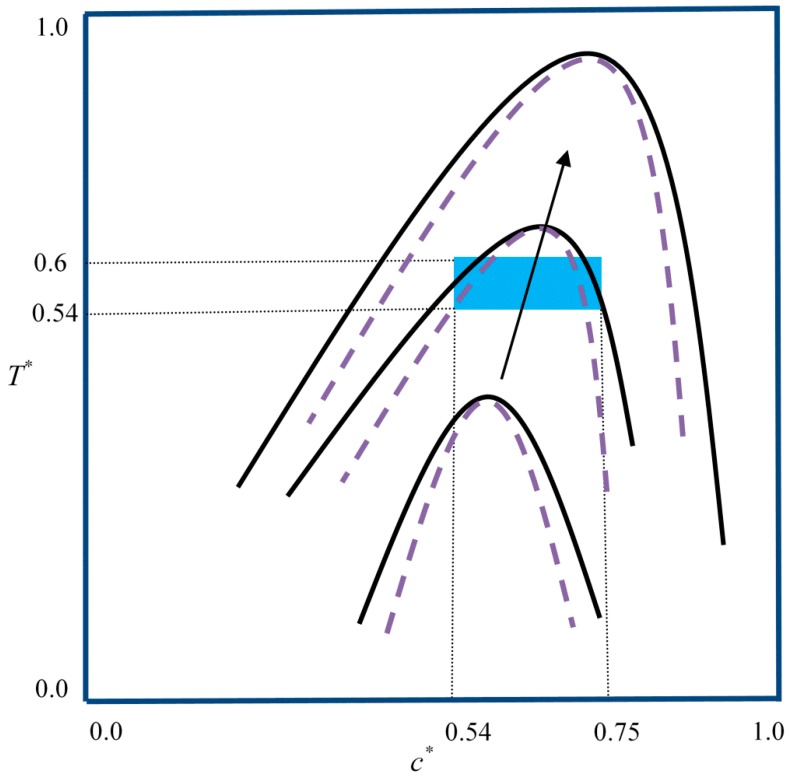
Schematic phase diagram of a binary mixture undergoing the polymerization-induced phase separation (PIPS) phenomenon. The symmetric phase diagram represents the initial state when the degrees of polymerization of both of the components are one. The phase diagram shifts upward to higher concentrations and becomes asymmetric with increasing polymer molecular weight through polymerization. Eventually, the sample is thrust into the unstable region and starts to phase separate. The blue box represents the domain in which all of the eight cases presented in this paper are located.

Figure 1 shows a schematic of the phase diagram of a binary system undergoing the PIPS process. The blue box indicates the range of all eight samples. The solid curve indicates the binodal curve, while the dash curve shows the spinodal line at different degrees of polymerization. The phase diagram is symmetric at the beginning of the process as the degrees of polymerization of both the solvent and solute are equal ($N_{1}$ = $N_{2}$ =1). As the polymerization reaction proceeds, the molecular weight of the polymer increases, and the equilibrium curve is elevated toward a higher concentration and temperature; it then gradually passes the curing point. Therefore, the sample is thrust into the unstable region of the phase diagram, and phase separation is induced by a spinodal decomposition mechanism. The rate of the upward movement of the phase diagram depends on the rate constant of the polymerization reaction as the degree of polymerization of the polymer ($N_{2}$) increases faster at a higher rate constant, which, in turn, depends on the temperature of the system. In addition, the initial concentrations of the components affect the polymerization lag time, which is the time it takes for the curing point to move into the unstable region.

**Figure 2:**
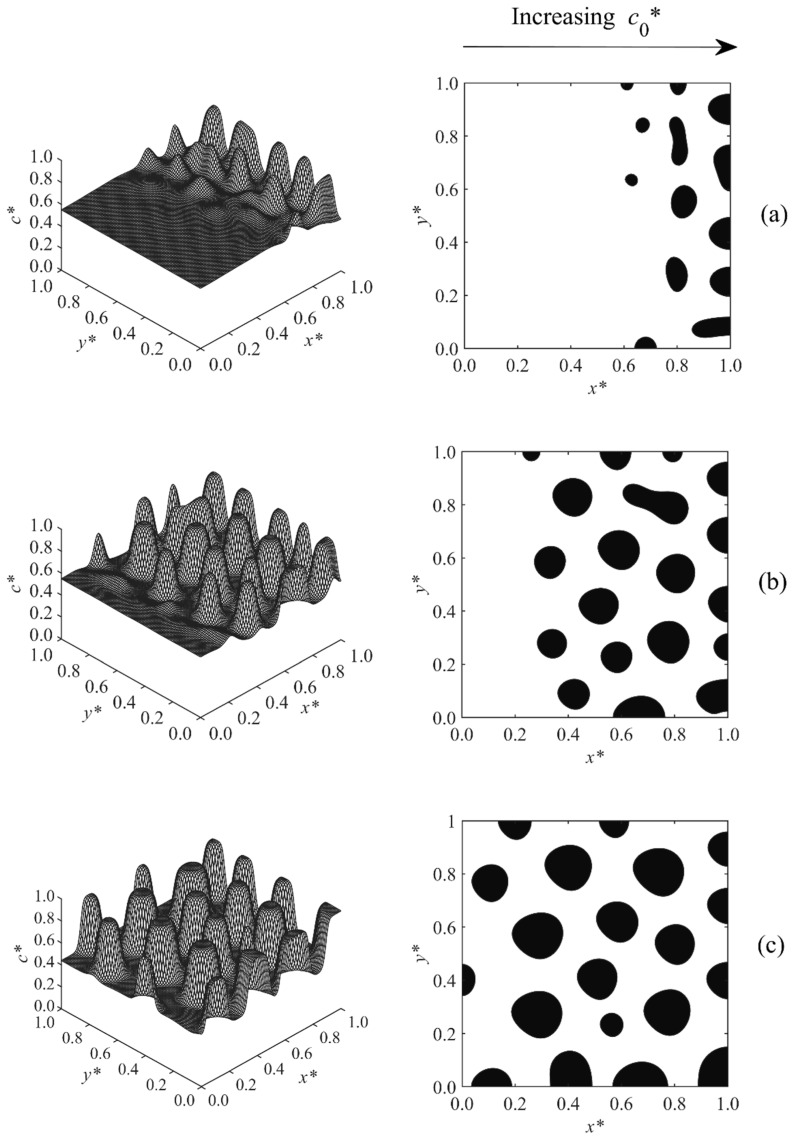
Spatial concentration profiles (first column) and phase-separated patterns (second column) for Case 1 at the following times: (**a**) $t^{*}$ = 2.14 × 10^−4^, (**b**) $t^{*}$ = 2.18 × 10^−4^, and (**c**) $t^{*}$ = 2.24 × 10^−4^.

**Figure 3:**
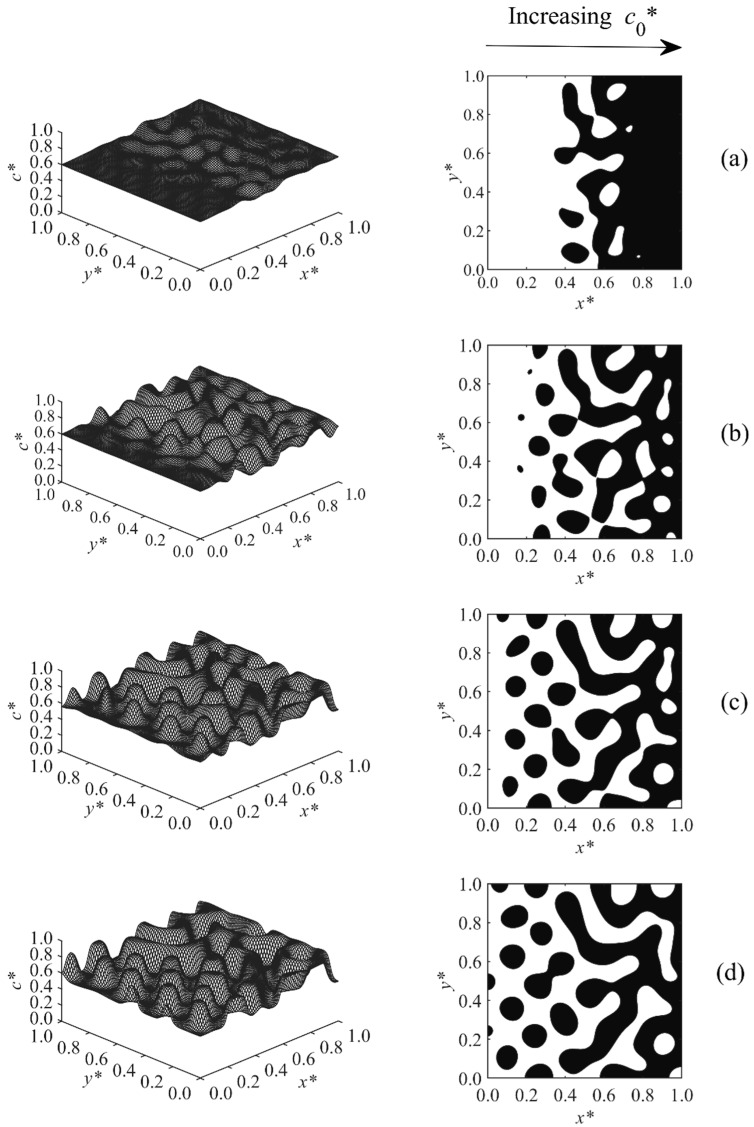
Spatial concentration profiles (first column) and phase-separated patterns (second column) for Case 2 at the following times: (**a**) $t^{*}$ = 9.8 × 10^−5^, (**b**) $t^{*}$ = 9.9 × 10^−5^, (**c**) $t^{*}$ = 1.0 × 10^−4^, and (**d**) $t^{*}$ = 1.01 × 10^−4^.

**Figure 4:**
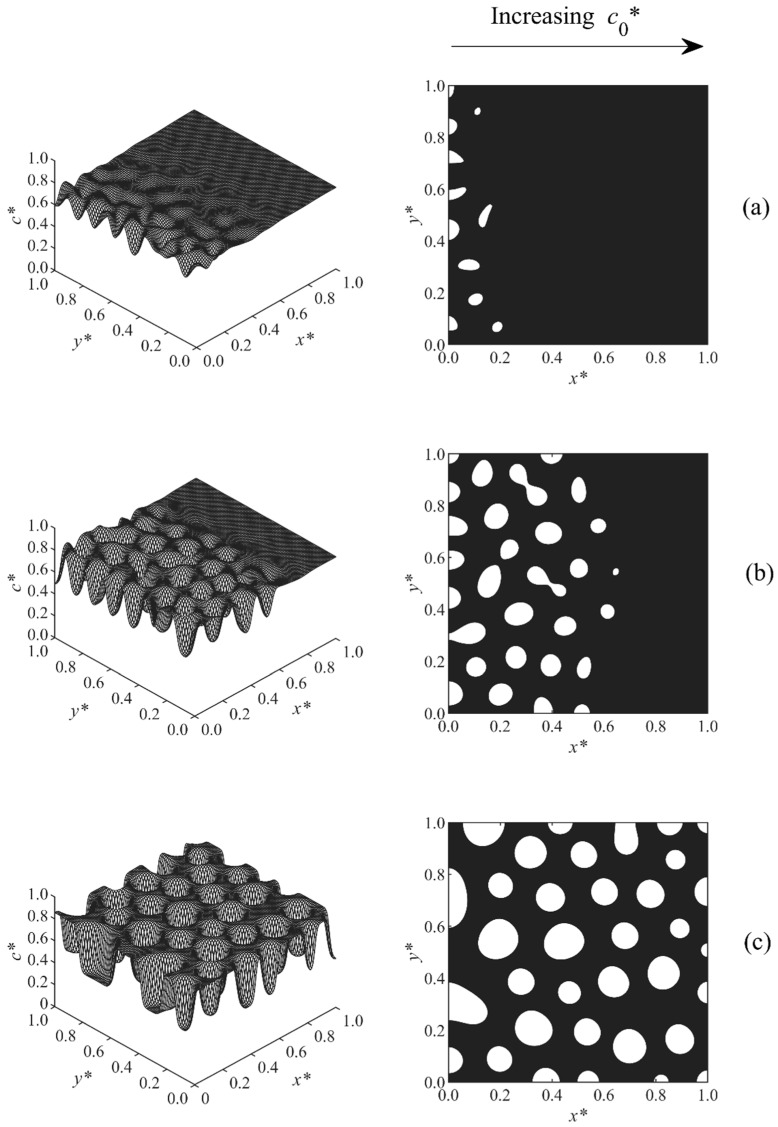
Spatial concentration profiles (first column) and phase-separated patterns (second column) for Case 3 at the following times: (**a**) $t^{*}$ = 1.01 × 10^−4^, (**b**) $t^{*}$ = 1.02 × 10^−4^, (**c**) $t^{*}$ = 1.07 × 10^−4^.

**Figure 5:**
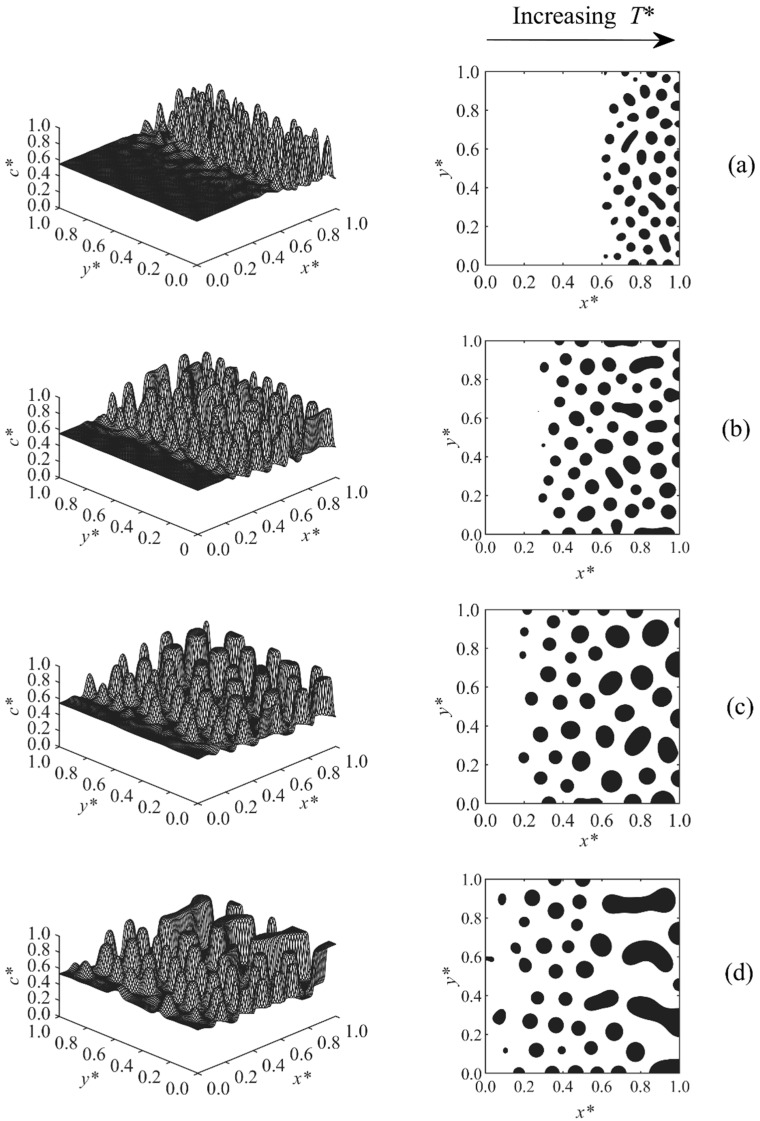
Spatial concentration profiles (first column) and phase-separated patterns (second column) for Case 4 at the following times: (**a**) $t^{*}$ = 4.312 × 10^−4^, (**b**) $t^{*}$ = 4.32 × 10^−4^, (**c**) $t^{*}$ = 4.34 × 10^−4^, and (**d**) $t^{*}$ = 4.37 × 10^−4^.

**Figure 6:**
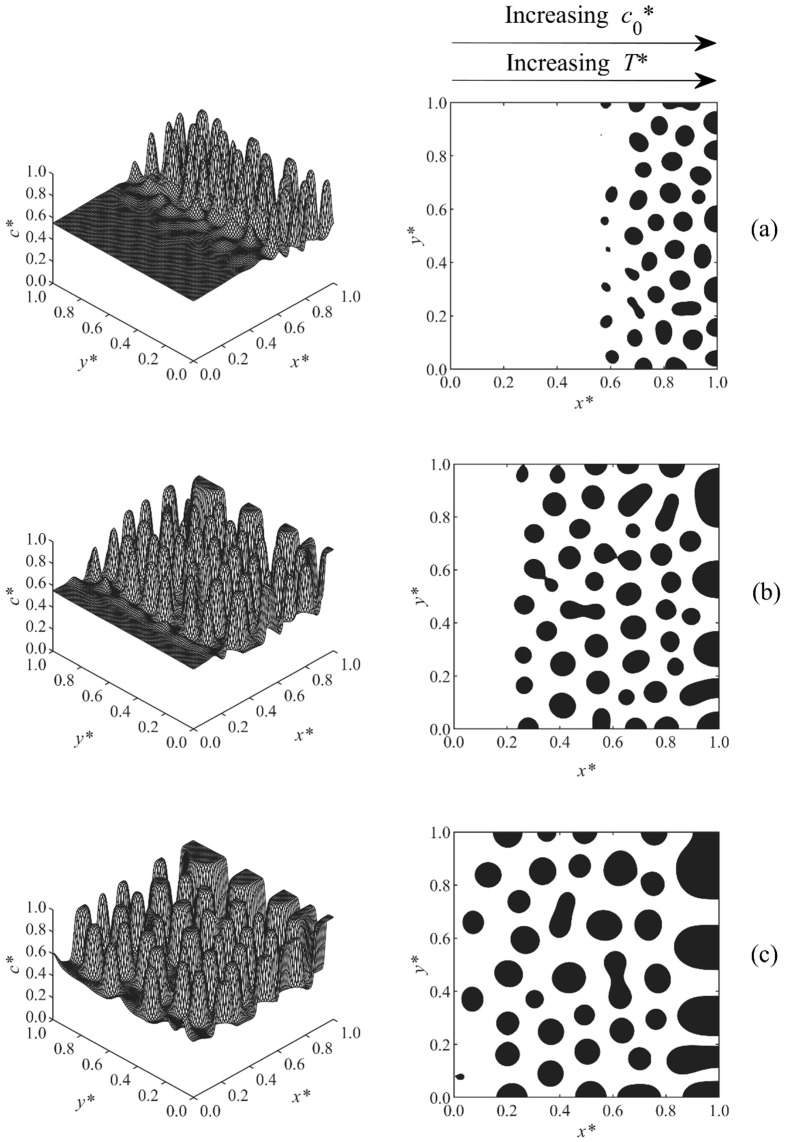
Spatial concentration profiles (first column) and phase-separated patterns (second column) for Case 5 at the following times: (**a**) $t^{*}$ = 5.93 × 10^−5^, (**b**) $t^{*}$ = 6.0 × 10^−5^, and (**c**) $t^{*}$ = 6.1 × 10^−5^.

**Figure 7:**
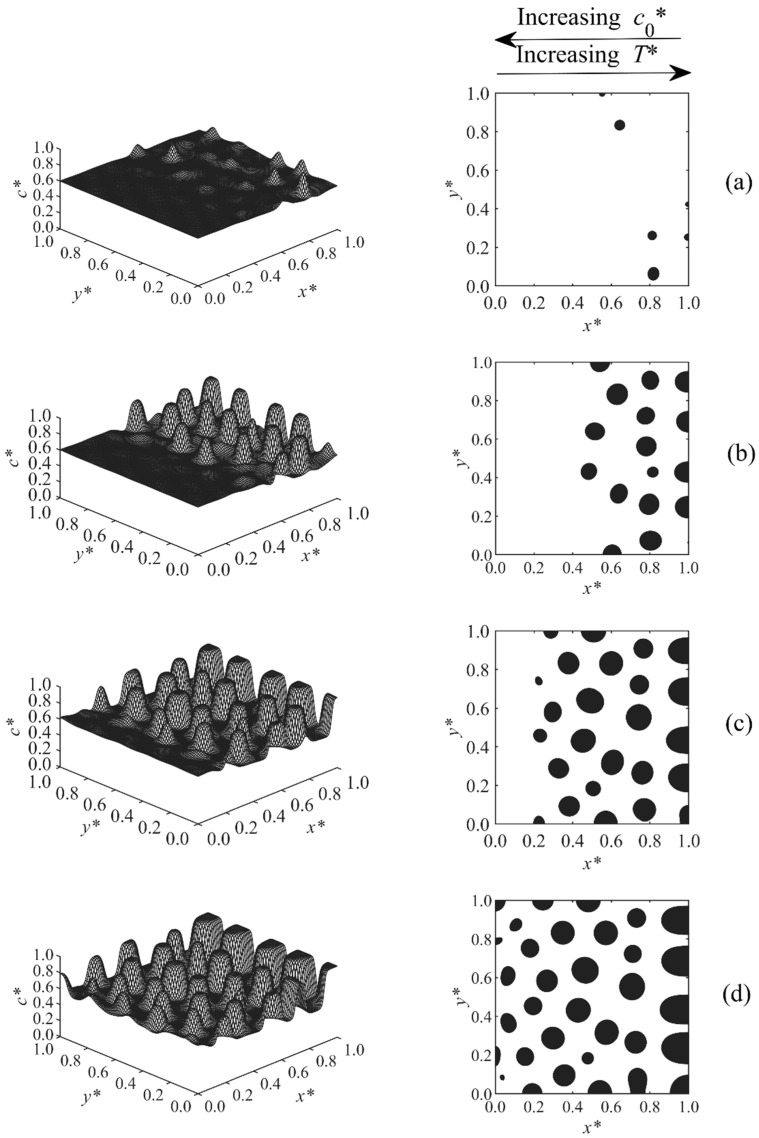
Spatial concentration profiles (first column) and phase-separated patterns (second column) for Case 6 at the following times: (**a**) $t^{*}$ = 1.095 × 10^−4^, (**b**) $t^{*}$ = 1.1 × 10^−4^, (**c**) $t^{*}$ = 1.11 × 10^−4^, and (**d**) $t^{*}$ = 1.12 × 10^−4^.

**Figure 8:**
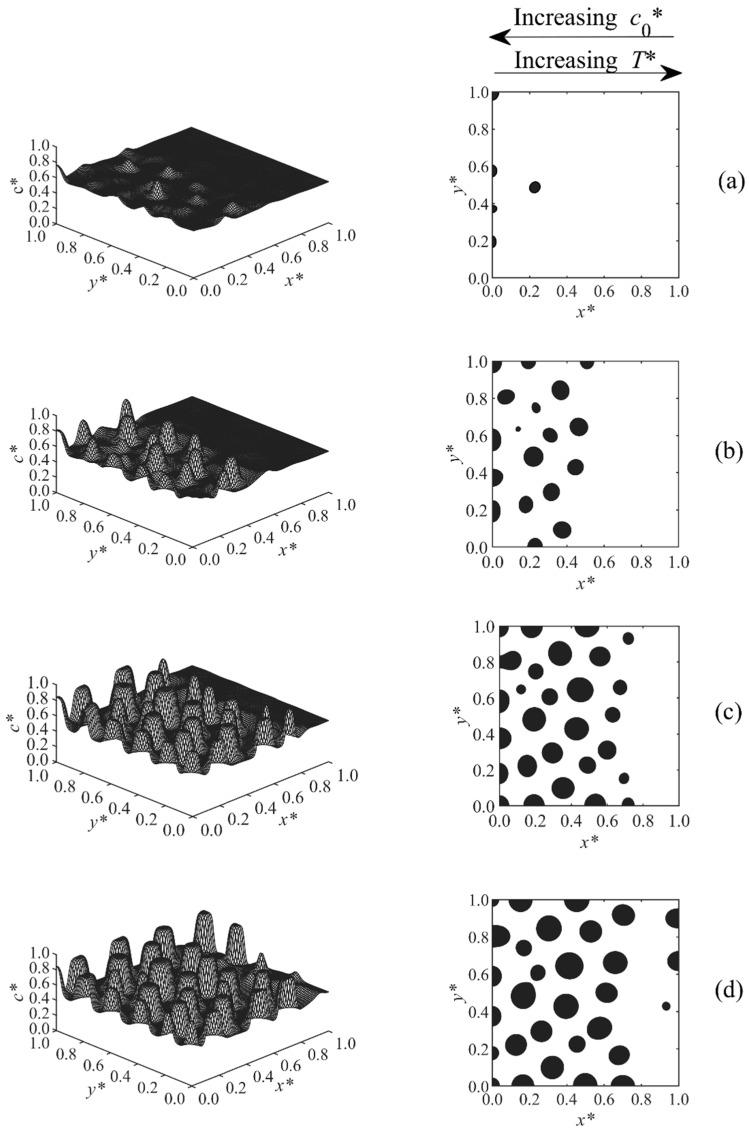
Spatial concentration profiles (first column) and phase-separated patterns (second column) for Case 7 at the following times: (**a**) $t^{*}$ = 5.66 × 10^−5^, (**b**) $t^{*}$ = 5.7 × 10^−5^, (**c**) $t^{*}$ = 5.8 × 10^−5^, and (**d**) $t^{*}$ = 5.9 × 10^−5^.

**Figure 9:**
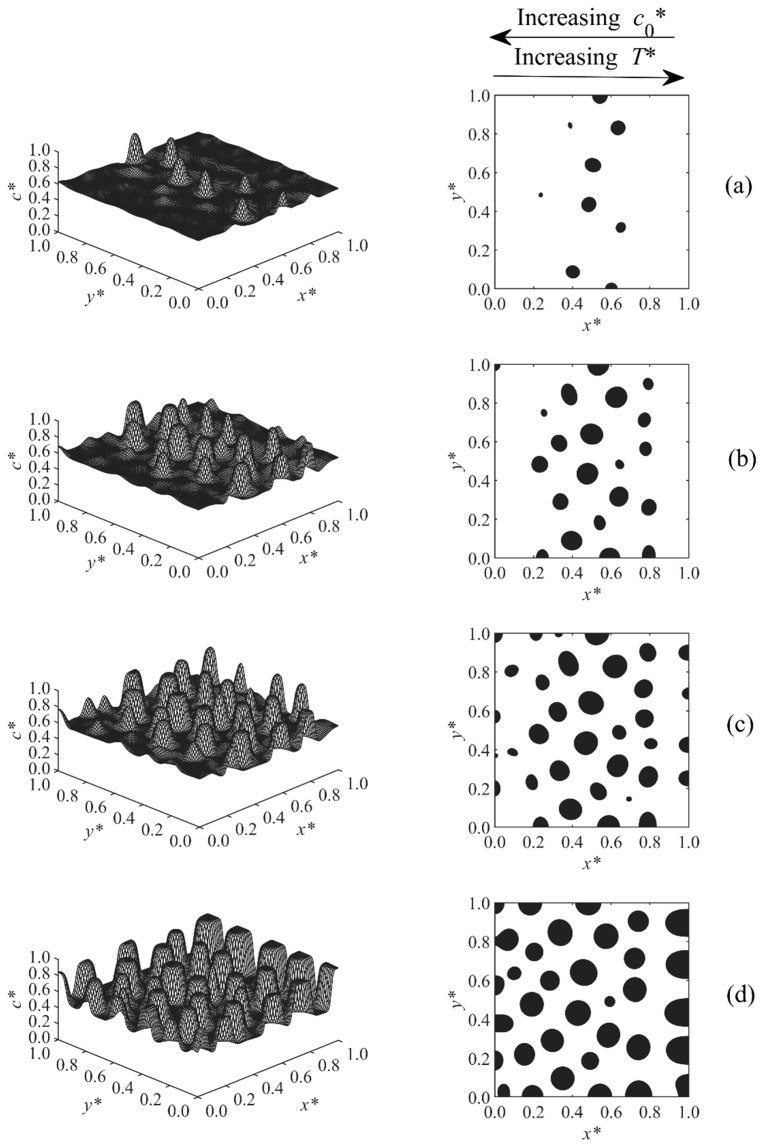
Spatial concentration profiles (first column) and phase-separated patterns (second column) for Case 8 at the following times: (**a**) $t^{*}$ = 5.62 × 10^−5^, (**b**) $t^{*}$ = 5.65 × 10^−5^, (**c**) $t^{*}$ = 5.67 × 10^−5^, and (**d**) $t^{*}$ = 5.8 × 10^−5^.

Figure 2, Figure 3, Figure 4, Figure 5, Figure 6, Figure 7, Figure 8 and Figure 9 below show the phase separated patterns for the eight cases listed in Table 1. As the initial concentration in all of the cases was in the range of 0.54–0.75, the average value of 0.645 was used as the boundary between black and white areas. The black area denotes the solvent-rich region (where the concentration of the solvent is greater than 0.645), while the white region is polymer-rich ($c_{o}{}^{*}$ < 0.645).

Figure 2 demonstrates the temporal evolution of the droplets for Case 1. In this case, the initial concentration of the solvent linearly changed in the range of $c_{o}{}^{*}=$ 0.55–0.6 across the sample while the temperature was fixed at $T^{*}=$ 0.6. The results indicate that the sample entered into the unstable region such that it was to the left of the critical region, since a solvent-rich droplet-type morphology was observed. The particles first appeared at the higher concentration section and eventually propagated to the lower concentration layers. The reason is that the polymerization lag time—the time it takes for the sample to enter into the unstable region of phase diagram—was inversely proportional to the initial concentration of the sample. The lag time decreased with increasing initial concentration, which means that the layer of high initial concentration entered into the unstable region and was phase-separated earlier than that of low concentration.

In Case 2, shown in Figure 3, the initial concentration gradient was set as $c_{o}{}^{*}=$ 0.6–0.7 and temperature $T^{*}=$ 0.6. While the polymerization reaction occurred and the thermodynamic curves shifted toward higher concentrations, the sample was thrust into the spinodal region in a way that it spanned across both the off-critical and the critical regions. This means that one portion of the sample was located in the left side of the critical area, one portion was located in the right side of the critical region, and another one was placed in the critical region. As Figure 3 shows, a complex morphology was reached, including an interconnected morphology in the middle layers and solvent-rich and solute-rich droplets at the left and right sides of the domain, respectively. As time proceeded, the equilibrium curve moved toward higher concentrations and temperatures which shifted the critical point toward higher concentrations. Therefore, the interconnected morphology shifted toward the right side of the domain while the droplet-type morphology grew in the left part as the lower concentration area was in the off-critical region.

In Case 3, shown in Figure 4, the initial concentration gradient was $c_{o}{}^{*}=$ 0.7–0.75. This concentration range was completely located right to the critical area in the unstable region. Therefore, the resultant droplets were rich with respect to the polymer (the polymer droplets were white while the continuous solvent matrix was black). In this case, the polymer droplets were observed earlier in the lower concentration region and afterward expanded to the higher concentration region. An asymmetric structure was observed along the concentration gradient with larger (smaller) droplets at the lower (higher) concentration side. The results of Figure 2, Figure 3 and Figure 4 are consistent with the results previously obtained for the TIPS process of polymeric solutions under a concentration gradient [31,32]. The phase diagram, however, is fixed in the TIPS studies, and the critical region does not shift, so the study of morphology development is simpler compared to the cases in this paper in which the quench depth and the location of the samples, with respect to the critical point, were continuously changing.

The phase-separated structure of Case 4 is described in Figure 5. In this case, only a linear temperature gradient $T^{*}=$ 0.54–0.55 was applied along the sample. The initial concentration was kept constant at $c_{o}{}^{*}=$ 0.55. An anisotropic morphology with larger particles at the higher temperature side and smaller droplets at the lower temperature side was achieved. The asymmetry was formed due to the faster upward shifting of the phase diagram with increasing temperature as the reaction rate constant was exponentially proportional to the temperature. Therefore, the phase diagram passed the region of high temperature earlier, so phase separation was induced, and the sample entered the early, intermediate, and even late stage of phase separation earlier and faster; this led to the larger droplets at the high temperature side. The droplets in the higher concentration region, $x^{*}=0.8-1.0$, coarsened at $t^{*}=4.37\times 10^{-4}$, indicating that this region was entering the late stage of phase separation. The results replicate the previous modeling results of the PIPS phenomenon of a polymer solution/blend imposed with a temperature gradient [34,35].

A linear initial concentration gradient coupled with a linear temperature gradient in the same direction was imposed to the sample of Case 5; the results are presented in Figure 6. A higher temperature led to the faster elevation of phase diagram, as the polymerization rate constant was exponentially proportional to the temperature; therefore, the phase diagram passed the sample with the higher temperature side faster, and phase separation was induced earlier. A higher concentration also led to a shorter polymerization lag time, and, thus, the higher concentration region started to phase separate earlier. As the directions of the concentration and temperature gradients were the same in Case 5, they amplified the effect of each other so larger particles first appeared at the high concentration and temperature edge, and smaller droplets appeared later at the lower side. As a result, a pronounced anisotropic structure was found. As Figure 6c illustrates, the particles in the higher temperature and concentration region reached the late stage and coarsened first. The reason is that at the higher concentration and temperature region, the sample entered the unstable region earlier, allowing it to go through phase separation for a longer period of time and reach the late stage while the lower concentration and temperature layers are still in the early or intermediate stages.

Cases 6–8 are shown in Figure 7, Figure 8 and Figure 9. In these cases, the concentration and temperature gradients were applied in opposite directions. The study of morphologies was complicated since there was a competition between the concentration and temperature effects. The temperature range $T^{*}=$ 0.595–0.6 was fixed for all three cases. Three different concentration ranges were used for these three cases, which resulted in three different morphologies.

In Case 6, shown in Figure 7, the range of initial concentration was $c_{o}{}^{*}=$ 0.56–0.6, the effect of temperature was dominant, so the growth of the particles was faster at the higher temperature and lower concentration side (the right side). Since the polymerization lag time is a function of both temperature and concentration, the combination of these two parameters resulted in a shorter or longer lag time. In this particular case, the lag time at initial concentration $c_{o}{}^{*}=$ 0.56 and temperature $T^{*}=$ 0.6 (right side of the sample) was shorter than that on the left side of the sample where $c_{o}{}^{*}=$ 0.6 and $T^{*}=$ 0.595. This resulted in the region of high temperature and low initial concentration entering the unstable region and phase separation earlier than the region of low temperature and high initial concentration.

In Case 7, shown in Figure 8, the range of concentration gradient was $c_{o}{}^{*}=$0.54–0.6. In this case, phase separation developed first at the higher concentration side (left side) and then moved toward the higher temperature region (right side), indicating that, at the initial concentration $c_{o}{}^{*}=$ 0.6 and temperature $T^{*}=$ 0.595, the polymerization lag time was smaller than that on the right side of the sample where $c_{o}{}^{*}=$ 0.54 and $T^{*}=$ 0.6. Therefore, the region of higher concentration and lower temperature (left side) entered the unstable region first and went through phase separation stages earlier and faster than the right side. As a result, larger particles were observed at the left side of the domain where the particles were undergoing phase separation longer. Since the lag time is inversely proportional to both temperature and initial concentration, their effects compete together. The effect of high initial concentration cancelled the effect of high temperature in this particular case, since they were increasing in opposite directions.

Figure 9 demonstrates the morphology development of Case 8, in which the concentration and temperature gradients were set in a way that the middle temperature and concentration layer entered into the unstable region earlier; therefore, the particles first grew in the middle and then expanded to both sides where the temperature and concentration were maximum.

### 3.2. Size and Morphology Analysis

**Figure 10:**
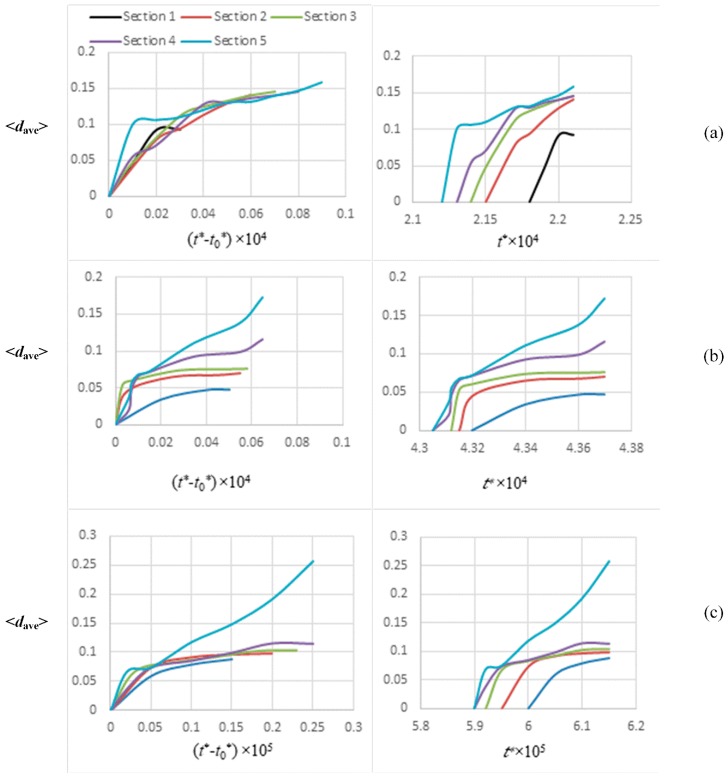
The average equivalent diameter of droplets developed within the five sections of a sample. Figures in rows **a**, **b**, and **c** represent Cases 1, 4, and 5, respectively. The left column is scaled according to t^*^–*t*_0_^*^, where *t*_0_^*^ is the polymerization lag time; the right column is scaled based on the t^*^.

As some of the cases studied in this work demonstrated complex morphological development due to the combination of the gradients applied in opposite directions or the upward movement of the phase diagram during polymerization, the asymmetric structural development of the samples were only investigated for Cases 1, 4, and 5. ImageJ software [44] was used to find the average equivalent diameter <*d*_ave_> of the droplets. The method of exclusion [45] was utilized to assess the size development of the three cases mentioned above. The domain was divided into five equal intervals; sections 1–5 from left to right. The particles located on the boundary of two sections were considered in the right region. The particles located on the boundary of the system were assumed as half droplets. The results are presented on Figure 10.

Figure 10 shows the average equivalent diameter growth with time for all the three cases of PIPS, including the concentration gradient (Case 1), temperature gradient (Case 4), and both gradients in the same direction (Case 5). The exponential growth coincided with the early stage of phase separation, while the slower growth after the exponential increase coincided with the intermediate and late stages of phase separation. The intermediate stage of phase separation and the late stage in some sections were studied. The right column of the figure indicates that for all of the cases, particles grew in the 5^th^ section, the section facing a higher concentration/temperature earlier while they grew later from section 4 to section 1. Growth in the lower sections occurred later due to a higher process induction time and slower elevation of phase diagram at the lower concentration/temperature. Therefore, the particles of different sections entered the unstable region and went through the early, intermediate, and late stages of the phase separation at different times.

Figure 10a demonstrates that the average diameter of the particles was larger in section 5, and it decreased from section 5 to section 1. The difference between the average diameters of various sections was more noticeable at early times, while the sizes in all the sections got close together at later times. The reason is that at earlier times ($t^{*}$ < 2.15 × 10^−4^), sections 4 and 5 were in the intermediate stage of phase separation while the others were still in the early stage of phase separation (section 3) or in the stable one phase region (sections 1 and 2). Therefore, the average diameters in different sections were different, and the morphology was anisotropic. At the later time ($t^{*}$ > 2.19 × 10^−4^), however, the droplets in all of the sections were in the intermediate stage of phase separation, so the growth rates and the average sizes got close together. In addition, when the time proceeded, the quench depth was continuously increasing, and the samples were getting far from the critical point; as such, at the higher sections, which undergo the phase separation longer, the growth rate of the particles decreased because they had reached the late stage of phase separation. This let the droplets at the lower sections grow and their diameters get close to those at the higher sections.

Figure 10b shows the corresponding results of Case 4, where just a linear temperature was applied to the sample. As expected, at a given instant, the average size was greater in section 5 while being smaller in the lower sections. The reason is that section 5 (high temperature region) entered the unstable region earlier due to the shorter process induction time and faster phase diagram elevation compared to the sections with lower temperature. Consequently, this section was imposed to the phase separation longer, and particles were larger at a given time. In sections 4 and 5, the diameters drastically grew at the late times, which show that the particles reached the late stage of phase separation.

Figure 10c shows that the growth rate of the diameters with time was much sharper compared to Figure 10a,b; this is reasonable, as a combined temperature and concentration gradient was applied to the system in the same direction (Case 5). This amplified the effect of each other, which resulted in faster growth and larger particles appearing. The late stage of phase separation in section 5 is recognizable, as the average diameter grew sharply at later times, indicating that the droplets coarsened. Lastly, in the intermediate stage of phase separation, the average diameter of the various sections for these three cases followed the power law $<d_{ave}>\propto t^{*}{}^{\alpha }$, where α was in a range between $\frac{1}{3}$ and $\frac{1}{2}$; this is in agreement with previous work [46]. This means that the increase of droplet diameter with time follows a universal scaling in the intermediate stage of phase separation by spinodal decomposition and can be predicted using this power law.

## 4. Conclusions

The PIPS phenomenon of a solvent/monomer under external fields was numerically modeled. Based on the directions and extent of the concentration/temperature gradient applied to the system, eight asymmetric structures were observed. The results showed that the larger (smaller) particles were formed earlier in the higher (lower) concentration/temperature region as a result of a shorter (longer) process induction time. For the cases in which the temperature and initial concentration gradients were applied in opposite directions, their effects competed against each other and provided three different morphologies. The results were consistent with published data. The size analysis was carried out using digital imaging software. For three of the cases, the larger particles were found at the higher temperature/concentration side. In some sections, the size grew sharply at later times, showing the late stage of the phase separation. The equivalent diameter was found to be proportional to t^*α^ where *α* was found in a range between $\frac{1}{3}$ and $\frac{1}{2}$ for the cases studied, which was consistent with previous work.

## Figures and Tables

**Table 1:** The parameters are defined for the eight cases studied in the current work. For all cases, $E_{a}^{*}$ = 10 and ψ = 1.

Case	Condition	*T* ^*^	*c*_o_^*^	D	*A* ^*^
Case 1	• Δ*c*_o_* • Left to the critical region	0.6	0.55–0.6	2 × 10^5^	5 × 10^10^
Case 2	• Δ*c*_o_* • Across the critical region	0.6	0.6–0.7	4 × 10^5^	10^11^
Case 3	• Δ*c*_o_* • Right to the critical region	0.6	0.7–0.75	4 × 10^5^	10^11^
Case 4	• ΔT* • Left to the critical region	0.54–0.55	0.55	4 × 10^5^	10^11^
Case 5	• ΔT^*^+ Δ*c*_o_^*^ • Same Direction • Left to the critical region	0.595–0.6	0.55–0.6	4 × 10^5^	2 × 10^11^
Case 6	• ΔT^*^+ Δ*c*_o_^*^ • Opposite directions • Left to the critical region	0.595–0.6	0.56–0.6	4 × 10^5^	10^11^
Case 7	• ΔT^*^+ Δ*c*_o_^*^ • Opposite directions • Left to the critical region	0.595–0.6	0.54–0.6	4 × 10^5^	2 × 10^11^
Case 8	• ΔT^*^+ Δ*c*_o_^*^ • Opposite directions • Left to the critical region	0.595–0.6	0.552–0.6	4 × 10^5^	2 × 10^11^
